# Genetic dissection of quantitative and qualitative traits using a minimum set of barley Recombinant Chromosome Substitution Lines

**DOI:** 10.1186/s12870-018-1527-7

**Published:** 2018-12-07

**Authors:** Carla De la Fuente Cantó, Joanne Russell, Christine A. Hackett, Allan Booth, Siobhan Dancey, Timothy S. George, Robbie Waugh

**Affiliations:** 1 The James Hutton Institute, Invergowrie, Dundee, DD2 5DA UK; 2 Biomathematics and Statistics Scotland (BioSS), Invergowrie, Dundee, DD2 5DA UK

**Keywords:** Barley, RCSLs, SNPs, QTLs, Candidate genes

## Abstract

**Background:**

Exploring the natural occurring genetic variation of the wild barley genepool has become a major target of barley crop breeding programmes aiming to increase crop productivity and sustainability in global climate change scenarios. However this diversity remains unexploited and effective approaches are required to investigate the benefits that unadapted genomes could bring to crop improved resilience. In the present study, a set of Recombinant Chromosome Substitution Lines (RCSLs) derived from an elite barley cultivar ‘Harrington’ as the recurrent parent, and a wild barley accession from the Fertile Crescent ‘Caesarea 26–24’, as the donor parent (Matus et al. Genome 46:1010–23, 2003) have been utilised in field and controlled conditions to examine the contribution of wild barley genome as a source of novel allelic variation for the cultivated barley genepool.

**Methods:**

Twenty-eight RCSLs which were selected to represent the entire genome of the wild barley accession, were genotyped using the 9 K iSelect SNP markers (Comadran et al. Nat Genet 44:1388–92, 2012) and phenotyped for a range of morphological, developmental and agronomic traits in 2 years using a rain-out shelter with four replicates and three water treatments. Data were analysed for marker traits associations using a mixed model approach.

**Results:**

We identified lines that differ significantly from the elite parent for both qualitative and quantitative traits across growing seasons and water regimes. The detailed genotypic characterisation of the lines for over 1800 polymorphic SNP markers and the design of a mixed model analysis identified chromosomal regions associated with yield related traits where the wild barley allele had a positive response increasing grain weight and size. In addition, variation for qualitative characters, such as the presence of cuticle waxes on the developing spikes, was associated with the wild barley introgressions. Despite the coarse location of the QTLs, interesting candidate genes for the major marker-trait associations were identified using the recently released barley genome assembly.

**Conclusion:**

This study has highlighted the role of exotic germplasm to contribute novel allelic variation by using an optimised experimental approach focused on an exotic genetic library. The results obtained constitute a step forward to the development of more tolerant and resilient varieties.

**Electronic supplementary material:**

The online version of this article (10.1186/s12870-018-1527-7) contains supplementary material, which is available to authorized users.

## Background

Breeding programmes have successfully increased yield and quality traits of major cereals including barley [1, 2]. However, the domestication and breeding history of this ancient crop has drastically reduced the genetic diversity and current improvements in crop performance and yield have stagnated and reached a plateau [3]. Recently there has been renewed interest in wild germplasm collections as sources of beneficial alleles to mitigate the effects of reduced diversity and to ensure the future efficiency of agriculture under a changing climate [4–6].

Barley has a broad geographical distribution and because of its long history has adapted to a wide range of different environments [7, 8]. Over 460,000 accessions of *Hordeum* are preserved in gene banks worldwide, second only to rice (773,948) and wheat (856,168) (FAO 2010) and provide an enormous potential resource for allele mining. There have been many descriptive and comparative studies examining diversity in wild and landrace barleys, but few examples utilising the allelic diversity have been reported [3]. Importantly, crosses between cultivated and wild barleys are fertile and several research groups have developed experimental genetic stocks. These include introgression lines of chromosomal segments from the wild barley genome into domesticated barley [9–13] and multi-parental nested populations with wild donor genomes [14–16]. Several of these populations have been used to introgress favourable alleles from wild barley into breeding populations for resistance to various diseases and to improve agronomic traits under post-anthesis drought. For example, disease resistance [17, 18] and increased performance under drought and saline conditions [19, 20] have been associated with genetic polymorphisms for which wild alleles contribute favourable genotypic variation for plant growth and yield under stress. In addition, the introgression of novel exotic allelic variants has been found to constitutively improve economically important traits related with the malting characteristics of the crop [21] as well as key yield components such as grain weight and seeds per spike [9, 22, 23].

The utilisation of high-throughput genotyping platforms in some of these studies together with field trial data over several seasons and/or sites facilitated targeting gene-based molecular markers associated with interesting traits that could potentially be deployed in crop breeding programmes. Recently, Saade et al. [20] and Cu et al. [21] were able to identify diagnostic SNP markers for which wild barley allelic variants were associated with significant increases in crop yield performance in saline environments and malting quality traits such as α-amylase activity and fermentability, respectively. However, the identification and utilisation of novel exotic alleles for improving barley commercially still remains limited in relation to other cereal crops since its contribution is mostly restricted to experimental populations [4, 24]. Nonetheless, in other cereal crops such as rice and wheat, [24, 25], the use of wild relatives’ genomes has proved crucial in the release of new varieties with improved tolerance to biotic and abiotic threats.

Therefore, optimising the identification of exotic alleles for barley is still a major undertaking for most crop improvement programmes. The recent advances in genomic technologies and sequencing have resulted in the identification of millions of single nucleotide polymorphisms (SNPs) [6, 26], and an almost complete genome sequence [27] provides the necessary means to identify candidate genes underlying important quantitatively inherited traits. In this context, traditional introgression breeding schemes through marker-assisted backcrossing and selection need to be reconsidered in the contribution of genetic gains to the crop. Several rounds of backcrossed generations to reduce the effect of undesirable alleles and phenotyping of hundreds (in biparental crosses) or over 1000 (in multiparental crosses) lines in experimental trials can make these studies time consuming and labour intensive [4, 28, 29]. However, this approach offers the opportunity to test novel allele combinations derived from wide crosses and the detection of rare alleles associated with important traits that would not be detected in large collections of germplasm [30]. Furthermore, the establishment of unique homozygous lines simplifies the genetic dissection of complex traits since these can be measured across locations and/or growing seasons in a genetically stable germplasm [31].

The selection of minimum sets of introgression lines representing the entire genome of the wild donor parent has been suggested as an approach for simplifying the exploration of novel exotic allelic variation while overcoming the trade-offs with using large numbers of recombinant lines [32]. In barley, subsets of introgression lines have been defined for the main AB-populations derived from biparental crosses, which cover most if not all the donor genotype and these have been used to identify QTLs by simple statistical approaches, facilitating the rapid screening of genetic variation for traits requiring detailed phenotypic evaluations. For example, Schnaithmann and Pillen [33] and Naz et al. [34] identified QTLs for Nitrogen Use Efficiency (NUE) and root morphological traits in subsets of 28 and 72 ingrogression lines (BC_3_S_4:6_) respectively.

For the present study, we used the Recombinant Chromosome Substitution Lines (RCSLs) developed by Matus et al. [9]. This was one of the first AB-populations developed using barley as a model crop and several studies have shown the potential of the lines for exploring novel allelic combinations for malting traits as well as for drought resistance and agronomic traits [9, 35–37]. Considering these previous studies, our main goal was to effectively decipher the genetics underlying the variation for qualitative and quantitative traits using a minimum group of lines representing the genome of the exotic wild barley accession, Caesarea 26–24, in the genetic background of the elite North American spring malting barley, cv. Harrington, used as recurrent parent of the population. By using a suitable field experimental approach, we aimed to assess the effect of the donor genome in the performance of the elite parent. Morphological, developmental and agronomic aspects of the crop were evaluated aiming to determine the potential of the wild genome to contribute constitutive improvements to the crop as well as to its adaptability in the conditions of our experiment. Additionally, we attempted to identify chromosome regions explaining the phenotypic variation encountered by the design of a suitable marker-trait association analysis for this minimum group of lines. Finally, we pursued the identification of candidate genes underlying the qualitative and quantitative variation of traits using the newest and most complete reference sequence barley map.

## Results

### Genotypic characterisation of 28 RCSLs

**Fig. 1:**
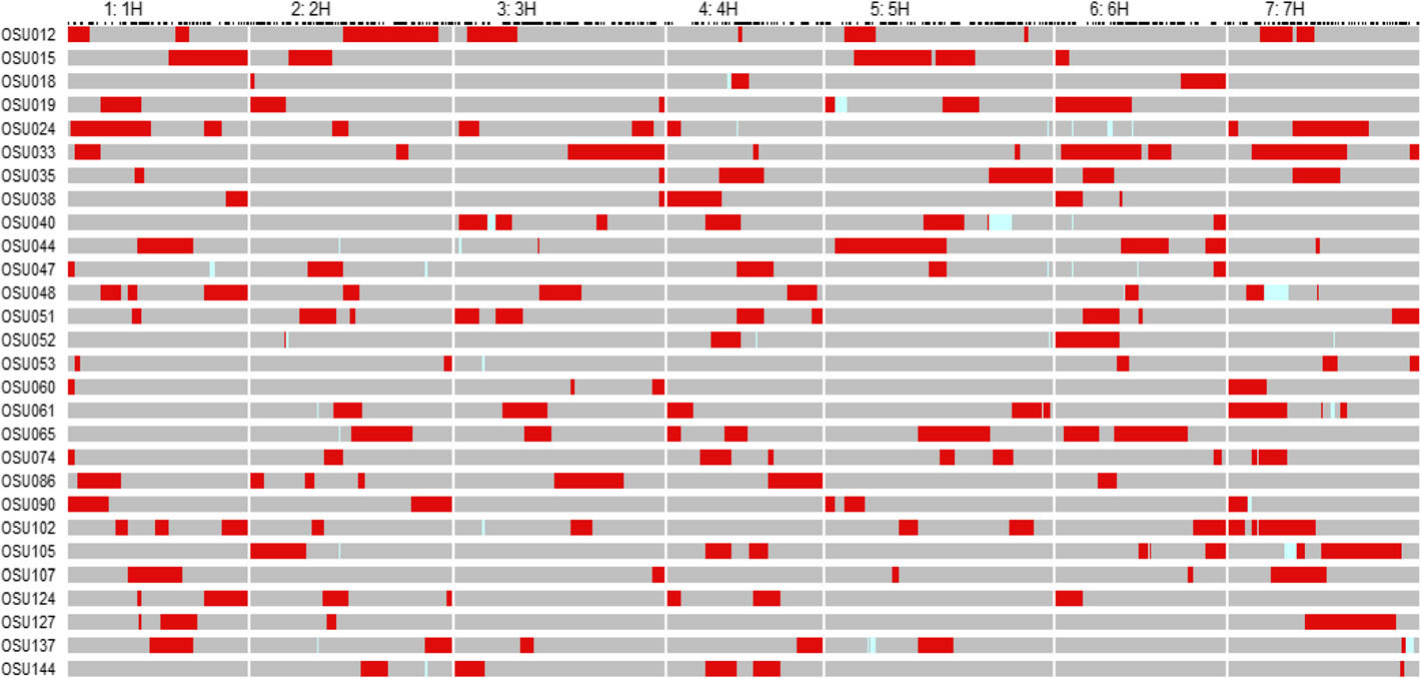
Graphical genotypes of 28 Recombinant Chromosome Substitution Lines (RCSLs) defined for 1848 polymorphic SNP markers. Caesarea 26–24 substituted segments in the RCSLs are represented in red; cv. Harrington genetic background in the RCSLs in grey. Missing marker data are indicated in light blue

The subset of 28 RCSLs, cv. Harrington and Caesarea 26–24 were successfully genotyped using the 7000 SNP-markers from the 9 K Infinium iSelect SNP platform [38]. The resulting genetic map consisted of 1848 SNP markers with a total length of 988.8 cM, with map positions determined using the Morex × Barke RILs population [38]. On average, the percentage of wild barley genome introgressed in the RCSLs corresponded to 13.4% but ranged from as low as 3.7% (OSU053) to 26.6% (OSU033) corresponding genetically from a few centiMorgans to almost half a chromosome (Fig. 1) (Additional file 1: Table S1).

In order to identify genomic regions associated with a range of phenotypic traits, the RCSLs were sown under a ‘rainout’ shelter specifically designed to deliver different amounts of water under the same experimental conditions. Over two seasons and contrasting water regimes 14 quantitative traits defining crop performance and 5 qualitative traits were scored (Table 1). Considerable variation was observed for all the measured traits, which were consistent across growing seasons and treatments, particularly for the qualitative traits. The effect of the water treatment and growing season significantly affected quantitative phenotypic scores, shaping the response of the lines for important morphological and agronomic traits as revealed by the Genotype by Treatment (GxT) and Genotype by Year (GxY) interaction effects (Table 2).

**Table 1:** Phenotypic traits scored in 2013 and 2014 field trials

Morphological traits			
Collar Height^a^	COL	cm	Plant height from soil surface to the main stem collar
Peduncle Length^a^	PdL	cm	Distance measured in the main stem from the first node to the collar
Peduncle Extrusion^a^	PdE	cm	Distance measured in the main stem from the flag leaf to the collar
Ear Length^a^	EAR	cm	Length of the ear excluding the awns
Seed Area^b, d^	SdA	mm^2^	Average area of the seed
Seed Length^b, d^	SdL	mm	Average length of the seed
Seed Width^b, d^	SdW	mm	Average width of the seed
Developmental traits			
No Tillers	TILL	tillers	Number of tillers harvested from the two middle rows of each plot
Heading date	HEA	DAS^c^	Number of days from sowing to 50% half of ear emergence (GS55) per plot
Agronomic traits			
Dry yield ^d^	DY	kg.ha^− 1^	Weight of the grain collected from two middle rows of each plot calculated for a hectare
Thousand grain weight^b, d^	TGW	g	Average weight of 1000 grains
Biomass yield^d^	BY	kg.ha^− 1^	Above ground dry biomass obtained from the two middle rows of each plot
Harvest Index^d^	HI	%	Ratio of dry yield to above ground dry biomass for the two middle rows of each plot calculated for a hectare
Grains per square meter^d^	GSM	grains.m^− 2^	Number of grains estimated for a square meter using TGW and DY values
Qualitative traits visually scored			
Spike glossiness	GLS		Presence/absence of glossy spike
Spike colour			Presence/absence of purple spikes
Seed shattering	SHT		Presence/absence of seed shattering
Grain threshability	THR		Presence/absence awn retention
Lodging	LOD		Presence/absence of lodging

^a^Heights were measured in three main stems from the two middle rows of each plot at harvest maturity (GS92)

^b^Seed morphological traits and TGW were determined by image analysis using MARVIN grain analyser

^c^*DAS* days after sowing

^d^Seed morphological traits and agronomic traits based on seed samples dried for 24 h at 100 °C

**Table 2:** Traits mean values (± SE) and maximum and minimum range values in parentheses for cv. Harrington (Harr) and the RCSLs under full irrigation (FI), partial irrigation (PI) and drought (DR) conditions in 2 years field trials (2013 and 2014). Overall mean values (± SE) for each treatment and year in italics. (a) Morphological traits (b) Developmental traits and (c) Agronomic traits

Trait (units)	2013			2014			
	FI	PI	DR	FI	PI	DR	Sig.^a^
2a							
COL (cm)							Y ns
Harr	93.2 ± 0.6	94.8 ± 2.0	83.4 ± 1.0	96.2 ± 1.7	97.3 ± 2.1	85.3 ± 0.5	T ^***^
	(91.3–94.0)	(90.7–100.0)	(81.0–85.7)	(92.3–100.3)	(92.0–102.0)	(84.0–86.3)	*G* ^***^
RCSLs	105.8 ± 1.0	107.7 ± 1.0	95.0 ± 0.9	106.5 ± 0.9	105.2 ± 1.0	95.2 ± 0.8	Y x T ns
	(78.0–137.7)	(87.0–136.3)	(70.3–122.3)	(83.0–136.0)	(81.3–137.3)	(80.3–125.0)	*Y x G* ^***^
Treatment	*105.4 ± 1.0*	*107.2 ± 0.9*	*94.6 ± 0.9*	*106.1 ± 0.9*	*104.9 ± 1.0*	*94.8 ± 0.8*	T x G ^***^
Year	*102.4 ± 0.6*			*101.9 ± 0.6*			Y x T x G ns
PdL (cm)							Y ^***^
Harr	31.4 ± 0.7	31.6 ± 1.2	28.0 ± 0.7	26.3 ± 1.6	28.9 ± 0.4	26.3 ± 2.0	T ^***^
	(30.0–33.0)	(28.3–33.7)	(27.0–30.0)	(22.7–30.3)	(28.0–30.0)	(22.0–30.0)	G ^***^
RCSLs	38.6 ± 0.4	39.4 ± 0.4	32.9 ± 0.4	32.9 ± 0.4	32.5 ± 0.4	29.3 ± 0.4	Y x T ^*^
	(31.0–50.3)	(28.7–64.0)	(24.0–43.7)	(20.7–45.3)	(23.3–44.7)	(20.7–40.3)	Y x G ^***^
Treatment	*38.4 ± 0.4 a*	*39.1 ± 0.4 a*	*32.7 ± 0.4 b*	*32.6 ± 0.4 a*	*32.3 ± 0.4 a*	*29.2 ± 0.4 b*	T x G ^**^
Year	*36.7 ± 0.3 A*			*31.4 ± 0.3 B*			Y x T x G ns
PdE (cm)							Y ^***^
Harr	11.8 ± 0.4	12.7 ± 1.4	8.8 ± 0.7	8.2 ± 1.1	8.9 ± 0.4	7.6 ± 1.7	T ^***^
	(11.0–12.7)	(8.7–15.0)	(7.3–10.7)	(5.7–10.7)	(8.0–9.7)	(3.7–10.3)	G ^***^
RCSLs	17.4 ± 0.3	18.2 ± 0.4	12.6 ± 0.4	12.9 ± 0.4	12.4 ± 0.3	10.0 ± 0.4	Y x T ^**^
	(10.3–28.0)	(8.0–39.0)	(5.0–23.0)	(1.7–25.7)	(3.7–23.0)	(1.3–19.3)	Y x G ^**^
Treatment	*17.2 ± 0.3 a*	*18.0 ± 0.4 a*	*12.5 ± 0.4 b*	*12.7 ± 0.4 a*	*12.3 ± 0.3 a*	*9.9 ± 0.4 b*	T x G ^**^
Year	*15.9 ± 0.2 A*			*11.7 ± 0.2 B*			Y x T x G ns
EAR (cm)							Y ^***^
Harr	8.8 ± 0.3	9.2 ± 0.2	7.7 ± 0.3	8.2 ± 0.4	8.6 ± 0.4	8.3 ± 0.4	T ^***^
	(8.0–9.7)	(8.7–9.7)	(7.0–8.3)	(7.3–9.3)	(7.7–9.3)	(7.3–9.0)	G ^***^
RCSLs	9.3 ± 0.1	9.3 ± 0.1	8.7 ± 0.1	8.9 ± 0.1	8.9 ± 0.1	8.8 ± 0.1	Y x T ^***^
	(6.7–12.0)	(7.3–13.0)	(7.0–10.7)	(7.0–11.3)	(6.3–13.0)	(6.7–12.7)	Y x G ^***^
Treatment	*9.3 ± 0.1 a*	*9.3 ± 0.1 a*	*8.7 ± 0.1 b*	*8.8 ± 0.1*	*8.9 ± 0.1*	*8.8 ± 0.1*	T x G ns
Year	*9.1 ± 0.1 A*			*8.8 ± 0.1 B*			Y x T x G ^*^
SdA (mm2)							Y ^***^
Harr	22.2 ± 0.1	22.3 ± 0.1	22.8 ± 0.2	20.7 ± 0.8	21.2 ± 0.6	21.2 ± 0.7	T ^*^
	(21.9–22.4)	(22.1–22.7)	(22.3–23.0)	(19.6–22.9)	(20.0–22.3)	(19.9–23.1)	G ^***^
RCSLs	22.9 ± 0.2	23.8 ± 0.1	24.2 ± 0.1	22.3 ± 0.2	22.7 ± 0.2	22.3 ± 0.1	Y x T ns
	(19.6–29.3)	(21.1–30.1)	(22.0–29.1)	(19.0–29.1)	(19.0–28.9)	(19.0–26.8)	Y x G ^***^
Treatment	*22.9 ± 0.2*	*23.8 ± 0.1*	*24.2 ± 0.1*	*22.3 ± 0.2*	*22.7 ± 0.2*	*22.3 ± 0.1*	T x G ^*^
Year	*23.6 ± 0.1 A*			*22.4 ± 0.1 B*			Y x T x G ns
SdL (mm)							Y ns
Harr	7.8 ± 0.0	8.1 ± 0.0	8.1 ± 0.0	8.0 ± 0.2	8.0 ± 0.2	7.8 ± 0.1	T ^*^
	(7.7–7.9)	(8.0–8.2)	(8.0–8.1)	(7.7–8.5)	(7.6–8.3)	(7.6–8.1)	G ^***^
RCSLs	8.2 ± 0.1	8.7 ± 0.1	8.7 ± 0.0	8.5 ± 0.1	8.5 ± 0.1	8.3 ± 0.1	Y x T ^**^
	(6.9–12.0)	(7.6–12.7)	(8.0–10.9)	(7.0–12.2)	(7.1–11.7)	(7.3–11.2)	Y x G ^*^
Treatment	*8.2 ± 0.1 b*	*8.7 ± 0.1 a*	*8.6 ± 0.0 a*	*8.4 ± 0.1*	*8.5 ± 0.1*	*8.3 ± 0.1*	T x G ^**^
Year	*8.5 ± 0.0*			*8.4 ± 0.0*			Y x T x G ns
SdW (mm)							Y ^***^
Harr	3.7 ± 0.0	3.6 ± 0.0	3.7 ± 0.0	3.4 ± 0.0	3.5 ± 0.1	3.5 ± 0.1	T ns
	(3.6–3.7)	(3.5–3.6)	(3.6–3.7)	(3.3–3.5)	(3.3–3.6)	(3.4–3.7)	G ^***^
RCSLs	3.6 ± 0.0	3.6 ± 0.0	3.6 ± 0.0	3.5 ± 0.0	3.5 ± 0.0	3.5 ± 0.0	Y x T ^*^
	(3.3–3.8)	(3.3–3.8)	(3.4–3.9)	(3.2–3.8)	(3.3–3.8)	(3.3–3.9)	Y x G ^***^
Treatment	*3.6 ± 0.0*	*3.6 ± 0.0*	*3.6 ± 0.0*	*3.5 ± 0.0*	*3.5 ± 0.0*	*3.5 ± 0.0*	T x G ^*^
Year	*3.6 ± 0.0 A*			*3.5 ± 0.0 B*			Y x T x G ^***^
2b.							
TILL (tillers)							Y ^***^
Harr	169.0 ± 10.2	182.5 ± 10.1	120.8 ± 2.8	140.3 ± 10.8	160.8 ± 8.9	132.8 ± 13.7	T ^***^
	(153.0–197.0)	(167.0–212.0)	(116.0–129.0)	(121.0–169.0)	(145.0–183.0)	(112.0–171.0)	G ^***^
RCSLs	169.7 ± 2.5	177.6 ± 2.3	114.2 ± 1.6	144.5 ± 2.1	155.7 ± 2.1	128.6 ± 1.9	Y x T ^***^
	(105.0–245.0)	(110.0–243.0)	(69.0–157.0)	(70.0–202.0)	(105.0–207.0)	(75.0–191.0)	Y x G ^*^
Treatment	*169.7 ± 2.4 a*	*177.7 ± 2.3 a*	*114.4 ± 1.6 b*	*144.4 ± 2.0 b*	*155.9 ± 2.0 a*	*128.8 ± 1.8 c*	T x G ns
Year	*153.9 ± 1.9 A*			*143.0 ± 1.3 B*			Y x T x G ns
HEA (DAS)							Y ^***^
Harr	65.8 ± 0.3	65.0 ± 0.4	64.0 ± 0.4	60.5 ± 0.3	60.7 ± 0.3	60.3 ± 0.5	T ^**^
	(65.0–66.0)	(64.0–66.0)	(63.0–65.0)	(60.0–61.0)	(60.0–61.0)	(59.0–61.0)	G ^***^
RCSLs	64.9 ± 0.3	63.7 ± 0.2	63.4 ± 0.2	59.1 ± 0.2	59.2 ± 0.2	59.1 ± 0.2	Y x T ^*^
	(56.0–73.0)	(55.0–69.0)	(56.0–68.0)	(52.0–62.0)	(52.0–63.0)	(52.0–63.0)	Y x G ^***^
Treatment	*64.9 ± 0.2 b*	*63.8 ± 0.2 a*	*63.4 ± 0.2 a*	*59.1 ± 0.2*	*59.2 ± 0.2*	*59.1 ± 0.2*	T x G ^**^
Year	*64.0 ± 0.1 A*			*59.2 ± 0.1 B*			Y x T x G ns
2c.							
DY (kg/ha)							Y ^***^
Harr	4207.3 ± 204.7	4316.2 ± 97.3	3250.9 ± 164.1	2723.4 ± 238.6	3397.9 ± 167.6	3010.7 ± 302.2	T ^***^
	(3703.5–4705.5)	(4134.5–4526.0)	(3004.8–3725.0)	(2213.0–3354.3)	(3071.5–3794.6)	(2568.6–3894.8)	G ^***^
RCSLs	3653.9 ± 61.1	3909.5 ± 56.7	2846.1 ± 38.5	2735.6 ± 50.2	3090.8 ± 53.2	2629.2 ± 46.3	Y x T ^***^
	(2266.8–6064.8)	(2574.5–5727.0)	(1930.8–3845.3)	(1288.6–4234.3)	(1997.3–5072.3)	(1151.8–4294.2)	Y x G ns
Treatment	*3673.0 ± 60.0 b*	*3923.5 ± 55.3 a*	*2860.1 ± 38.1 c*	*2735.1 ± 49.0 b*	*3101.5 ± 51.9 a*	*2642.4 ± 46.1 b*	T x G ^***^
Year	*3485.5 ± 38.6 A*			*2825.5 ± 30.2 B*			Y x T x G ^*^
TGW (g)							Y ^***^
Harr	45.8 ± 0.4	45.0 ± 0.9	46.3 ± 0.9	38.9 ± 0.9	39.9 ± 0.9	46.6 ± 1.4	T ^***^
	(45.0–46.9)	(43.2–47.1)	(44.2–48.4)	(36.9–40.9)	(37.7–42.0)	(43.1–49.6)	G ^***^
RCSLs	45.9 ± 0.3	47.2 ± 0.3	48.1 ± 0.2	43.0 ± 0.3	44.0 ± 0.3	47.6 ± 0.3	Y x T ^***^
	(37.7–54.0)	(41.3–54.1)	(42.2–55.9)	(35.0–48.8)	(36.0–52.9)	(39.8–56.1)	Y x G ^***^
Treatment	*45.9 ± 0.3 b*	*47.1 ± 0.3 a*	*48.1 ± 0.2 a*	*42.8 ± 0.3 b*	*43.8 ± 0.3 b*	*47.5 ± 0.3 a*	T x G ^***^
Year	*47.0 ± 0.2 A*			*44.7 ± 0.2 B*			Y x T x G ^***^
BY (kg/ha)							Y ^***^
Harr	8862.5 ± 412.4	9131.3 ± 332.2	6793.8 ± 396.9	6225.0 ± 694.6	7787.5 ± 210.3	6700.0 ± 711.3	T ^***^
	(7875.0–9850.0)	(8350.0–9850.0)	(6150.0–7875.0)	(5075.0–7475.0)	(7400.0–8275.0)	(5650.0–8775.0)	G ^***^
RCSLs	8822.1 ± 107.5	9083.3 ± 107.9	6567.6 ± 82.9	6813.0 ± 123.3	7547.7 ± 106.1	6210.5 ± 88.9	Y x T ^***^
	(6025.0–13,450.0)	(6300.0–11,850.0)	(4350.0–8675.0)	(3925.0–10,350.0)	(5150.0–11,050.0)	(4250.0–10,425.0)	Y x G ns
Treatment	*8823.5 ± 104.5*	*9084.9 ± 104.6*	*6575.4 ± 81.0*	*6792.4 ± 121.2*	*7556.1 ± 102.7*	*6227.4 ± 88.8*	T x G ns
Year	*8161.3 ± 82.4*			*6862.7 ± 67.3*			Y x T x G ns
HI (%)							Y ^***^
Harr	47.5 ± 0.6	47.4 ± 0.9	47.9 ± 0.6	44.5 ± 0.4	43.6 ± 1.8	45.1 ± 2.0	T ^***^
	(46.2–48.9)	(45.9–49.9)	(46.6–49.0)	(43.6–45.0)	(39.7–47.5)	(39.8–48.8)	G ^***^
RCSLs	41.3 ± 0.4	43.0 ± 0.3	43.4 ± 0.3	40.2 ± 0.5	40.9 ± 0.4	42.4 ± 0.4	Y x T ns
	(27.9–48.8)	(33.1–50.3)	(35.4–51.2)	(28.8–50.2)	(32.0–48.3)	(19.0–51.0)	Y x G ^***^
Treatment	*41.5 ± 0.4 b*	*43.2 ± 0.3 a*	*43.6 ± 0.3 a*	*40.3 ± 0.5*	*41.0 ± 0.4*	*42.4 ± 0.4*	T x G ^**^
Year	*42.8 ± 0.2 A*			*41.4 ± 0.2 B*			Y x T x G ns
GSM (grains.m^2^)							Y ^***^
Harr	9170.2 ± 369.5	9606.0 ± 341.8	7029.4 ± 352.7	6985.4 ± 519.8	8521.3 ± 438.5	5856.4 ± 692.6	T ^***^
	(8233.7–10,037.3)	(8855.7–10,486.6)	(6203.0–7922.2)	(7528.2–9665.4)	(6000.5–8453.4)	(3539.6–7853.9)	G ^***^
RCSLs	7934.2 ± 118.9	8250.9 ± 128.5	5909.1 ± 86.0	6355.0 ± 116.9	7015.8 ± 106.7	5537.5 ± 95.8	Y x T ^***^
	(4785.4–11,667.5)	(5200.0–12,032.4)	(3183.2–8397)	(4397.4–10,896.5)	(3342.6–9060.9)	(2297.5–9341.2)	Y x G ns
Treatment	*7975.4 ± 117.2*	*8297.3 ± 126.6*	*5946.8 ± 85.8*	*6376.0 ± 114.4*	*7066.4 ± 106.9*	*5550.7 ± 95.6*	T x G ^***^
Year	*7403.085 ± 84.5*			*6329.0 ± 69.2*			Y x T x G ^*^

*ns* not significant

^***^P < 0.001, ^**^P < 0.01,^*^P < 0.05

^a^Statistically significant effect of the year (Y), water treatment (T), the genotype (G) and their interaction (YxT, YxG, TxG, YxTxG) on each trait performance. Statistical values (*p*-values) are provided for the fixed effects using a chi-squared based Wald-test using residual maximum likelihood (REML)

### Phenotypic variation and genetic dissection of qualitative traits

Five traits were measured visually, including glossy spike and colour, seed shattering, grain threshability and lodging, all of which are common characteristics of wild barley. The last three of these are undesirable traits for crop production and were observed in a few of the lines, OSU12 still shattered, OSU15 awns remained during threshing and OSU65 was prone to lodging. Interestingly, glossy spike, which may play a role in regulating water loss, was present in 5 of the RCSLs (OSU012, OSU047, OSU060, OSU074, and OSU090) and purple coloration of the spike was observed in OSU012, OSU065, and OSU144. For both spike characters we were able, looking at the overlapping exotic chromosome regions shared across groups of lines showing the same phenotype, to identify candidate regions for both traits. An association with a locus (GLS1) on top of chromosome 1H (0 to 3.2 cM), flanked by 12_30969 and 12_30715 was identified for glossy spike. From the pseudomolecules [27] we were able to physically define a 584,212 bp size region with 20 annotated genes. The annotation and descriptors of the genes in and around the locus were assessed as candidates potentially associated with the wax biosynthesis pathway. Three interesting candidates were identified based on its predicted function: an “esterase/lipase/thioesterase family protein” (HORVU1Hr1G000320.14) an “O-acyltransferase (WSD1-like) family protein” (HORVU1Hr1G000150.13) and a “Fatty acyl-CoA reductase 1” (HORVU1Hr1G000190.3). The last two were located at 551,441 bp and 453,531 bp from the position of the marker 12_30969. Similarly, a region associated with the purple coloration of the grain was defined on chromosome 2H (86 cM to 97.8 cM). The physical position of the flanking markers (SCRI_RS_116694 and SCRI_RS_153793) determined a 6.5 Mbp region with 215 associated transcripts. A “basic helix-loop-helix (bHLH) DNA-binding superfamily protein” (HORVU2Hr1G095870.1) and a “Myc-like anthocyanin regulatory protein” (HORVU2Hr1G096810.9) were identified as interesting candidate genes. Finally, chromosome regions represented by single RCSLs were found associated with known domestication traits such as the brittle spike (3H, 23.9 cM to 35.1 cM) and awn retention or reduced grain threshability (1H, 95.9 cM to 100.9 cM).

### Phenotypic variation and genetic dissection of quantitative traits

#### Morphological and developmental traits

The water treatment had a marked effect on plant growth. Overall, plants under drought were shorter than under irrigation. Considering collar height (COL) as total plant height, water stress reduced average plant height by 12.6 cm in 2013 and 10.1 cm in 2014 as compared with non-stress treatment (Table 2a). In this case the effect of the year was not significant, and the mean values reached were very similar across growing seasons. In regard to the length of the peduncle (PdL) and spike (EAR), these were significantly shorter in 2014 than in 2013. In addition, the water stress reduced peduncle length considerably both years, particularly in 2013 where the difference between non-stressed and stressed was 6.4 cm (3.1 cm in 2014). Furthermore, the spikes were significantly shorter under drought in 2013, with a slight difference of 0.6 cm as compared with the irrigated plots, but similar across water treatments in 2014. In general, the RCSLs were considerably taller than Harrington and showed longer peduncles and spikes.

Additionally, crop development varied across the trials (Table 2b). The 2014 growing season was significantly shorter than in 2013: plants reached heading 5 days earlier on average within a period of 10 days (18 days in 2013). The shortened vegetative growth could possibly explain the significant reduction in the number of tillers for all genotypes in 2014. Generally, the RCSLs flowered earlier than Harrington and developed fewer tillers.

#### Agronomic traits

Significant genotypic differences were observed for the scored agronomic traits across growing seasons. Both cv. Harrington and RCSLs genotypes performed better in 2013 than in 2014 showing overall dry yield (DY) values of 3485.5 kg ha^− 1^ and 2825.5 kg ha^− 1^, respectively (Table 2c). Similar observations were made for Thousand Grain Weight (TGW), biomass yield (BY), harvest index (HI) and grains m^2^ (GSM).

Large differences in crop performance, as measured by the agronomic traits, were also found as a consequence of the water treatment (*P* < 0.001). Dry grain yield, biomass and grains per m^2^ were negatively affected by water deficit showing a 27.1, 27.6 and 28.3% overall decrease in 2013, 14.8, 17.6 and 21.4% in 2014. Interestingly, despite droughted plots yielding less, the mean values for TGW tended to be larger under water stress (48.1 g in 2013 and 47.5 g in 2014) than in non-stress conditions (47.1 g in 2013 and 43.8 g in 2014), although these differences were only significant in 2014 with a 7.8% difference in the overall mean value (Table 2c). The partially irrigated treatment was considered as the control condition to measure the effect of drought in reference to a non-stressed water regime. These plots reached consistently greater yields across seasons providing a more appropriate potential yield value for the experimental conditions. An excess of moisture in the soil profile of fully irrigated plots appeared to be sub-optimal for crop growth and yield, particularly in 2014 growing season (with fully irrigated plots showing yield values in the range of the stressed plots (DY (kg/ha) of 1288.6–4234.3 compared to 1151.8–4294.2 for drought (Table 2c)). The greater accumulated rainfall and rainy days prior to the beginning of the experiment in 2014 could have had an effect on the increased groundwater moisture registered regularly throughout the growing season, particularly at the greater depth in the soil profile (i.e. 40 cm, Additional file 2: Figure S1 & Additional file 3: Figure S2)(Additional file 4: Table S2). For yield, the mean values for the RCSLs tended to be smaller than those for cv. Harrington in all water treatments (Table 2c). However, the range of variation in the RCSLs was greater, reaching maximum yield values in non-stressed plots of 6064.8. kg ha^− 1^ in 2013 and 5072.3 kg ha^− 1^ in 2014 to 3845.3 kg ha^− 1^ in 2013 and 4294.2 kg ha^− 1^ in 2014 under water stressed conditions (Table 2c). In contrast, the mean value for Thousand Grain Weight (TGW) was greater in the RCSLs compared to cv. Harrington in the three water treatments, with maximum weights of 55.9 g and 56.1 g under water stressed in 2013 and 2014 respectively (48.4 g and 49.6 g in cv. Harrington) (Table 2c).

A multi-comparison test using cv. Harrington dry yield and TGW estimated genotypic values as reference, showed that, even although none of the RCSLs had significantly improved yield performance, ten RCSLs (OSU018, OSU035, OSU040, OSU047, OSU052, OSU053, OSU060, OSU090, OSU107 and OSU144) showed estimated yield values comparable to the elite variety. Eight of these had significantly improved TGW means (OSU018, OSU040, OSU047, OSU052, OSU053, OSU060, OSU090 and OSU144) (Additional file 5: Table S3).

#### GE interaction and stability of agronomic traits

**Fig. 2:**
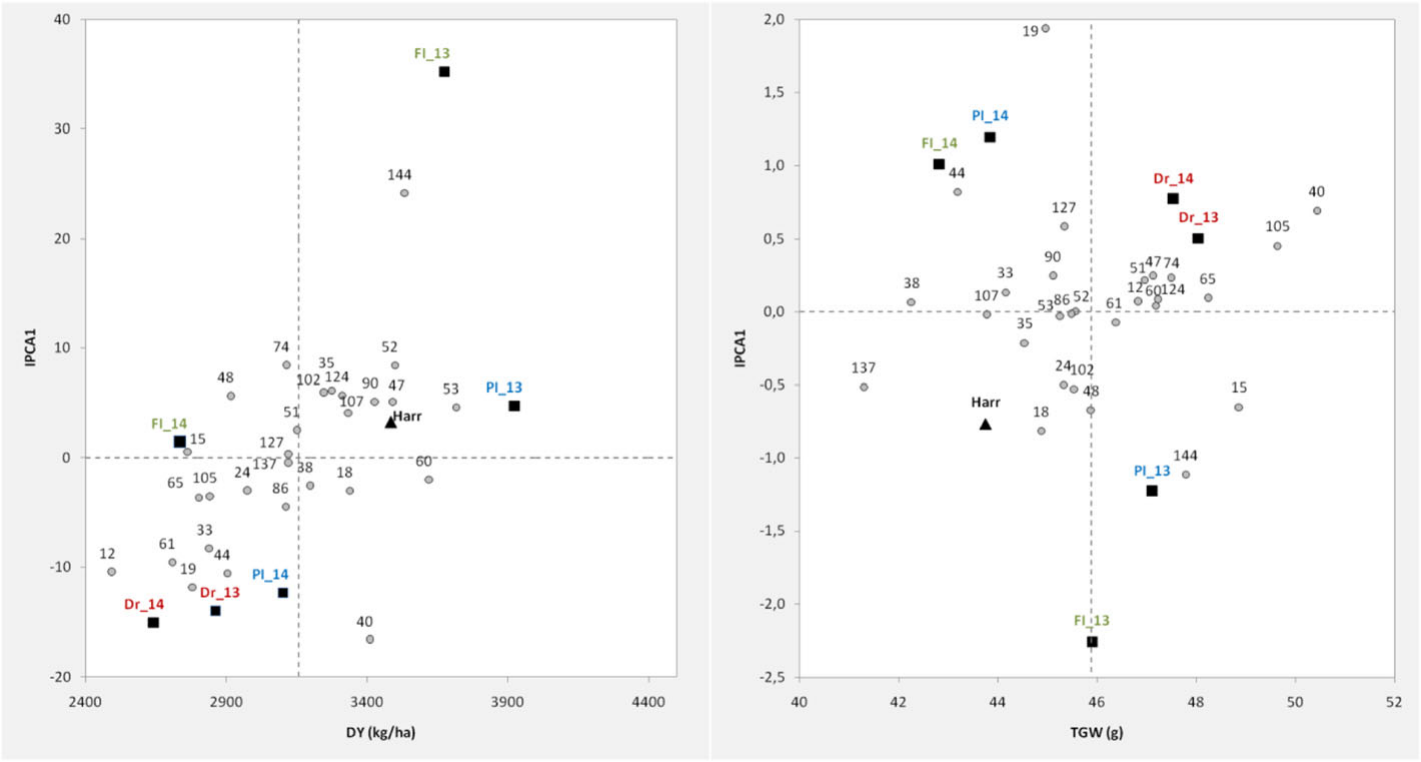
AMMI1 biplots for dry yield (left) and TGW (right) where the overall mean genotypic value for the trait (x axes) is represented against the IPCA1 score (y axes) of each line used as a measure of yield stability with the RCSLs and cv. Harrington. The analysis was conducted considering field trial data over three water treatments (FI, PI, DR) and 2 years (13, 14) (6 environments or year/treatment combinations). Genotypes score in grey numbered according to the RCSLs (OSU) number. Environments score in bold coloured capital letters

Because we identified significant variation for dry yield and TGW in response to the environmental conditions (growing season and water treatment), we used an AMMI analysis to interpret our findings further. For dry yield, the environment was the main source of variation explaining 44.1% of the model sum of squares, whereas genotype accounted for 17.6%. Conversely, the weight of the genotype on the sum of squares for TGW (32.8%) was larger than that for environments (29.3%). In addition, the GE interaction for dry yield was minor (9.3%) were overall not significant (*P* = 0.0682) as compared to the GE for TGW (10.4%, *P* < 0.001). Yet, the significant interaction with IPCA1 (*P* < 0.001) for dry yield, suggests slight differences in the genotypes specific adaptation for the trait. Nevertheless, the variation in the genotypes relative performance in different environments was more noticeable for grain weight than for dry yield (Table 3). These results are illustrated by the AMMI2 biplots which reflect the variation across genotypes in the GE interaction and the relationships between the environments (data not shown). However, we focused on the results of the AMMI1 biplots for dry yield and TGW for the identification of RCSLs with improved performance and stability in relation to the elite variety cv Harrington (Fig. 2). Harrington showed good yield performance and relatively stable for the conditions of the experiment, however its mean values for grain weight were lower and less stable than those for the majority of the RCSLs. Genotypes such as OSU047, OSU053 and OSU060 not only had genotype main performances in the range of Harrington but were broadly adapted to the environments tested showing more overall stability in TGW (IPCA1 close to zero).

**Table 3:** AMMI analysis of Dry yield (DY) and thousand grain weight (TGW) of RCSLs and Harrington across six environments (treatment/season combination)

Source of variation	d.f.	DY			TGW		
		s.s.	s.s.%^a^	*P*-value	s.s.	s.s.%^a^	*P*-value
Genotypes (G)	28	64,299,273	17.62	< 0.001	2830	32.83	< 0.001
Environments (E)	5	161,011,460	44.12	< 0.001	2612	29.27	< 0.001
Reps within E	18	5,202,435	1.43	0.1035	348	3.90	< 0.001
Interactions (GE)	140	33,908,173	9.29	0.0682	930	10.42	< 0.001
Partition of GE							
IPCA1	32	13,549,450	*39.96*	< 0.001	394	*42.37*	< 0.001
IPCA2	30	8,248,377	*24.33*	0.0895	226	*24.30*	0.0064
Residual	78	12,110,346	*35.72*	0.9141	310	*33.33*	0.5958
Error	504	100,495,504	27.54		2106	23.60	
Total	695	364,916,846			8925		

^a^Percentage of the model sum of squares for genotypes (G), environments (E) and the interaction GE; percentage of the GE sum of squares for IPCAs in italics

### QTL identification

A total of 177 stable marker-trait associations across experiments and treatments were identified for 14 quantitative traits (*P* < 0.05) using the mixed model association analysis that tested the effect of SNP polymorphisms on the performance of quantitative traits (Table 4). Eighty-five loci-trait associations were defined for the marker main effect (major QTLs) and 80 on its interaction with treatment (minor QTLs). On average, chromosome regions affecting the phenotype spanned 14.5 cM with the main effect peak QTL genomic region of 5.9 cM. The exotic genome was found to increase the traits’ mean performance in 55.3% of the loci associations compared to the 44.7% where the phenotypic mean was reduced. The wild alleles were associated with increases in the estimated means of morphological traits such as collar height and peduncle length whereas agronomic traits such as dry yield and harvest index showed diminished performance (Table 2; Additional file 6: Table S4).

**Table 4:** Quantitative traits loci summary table

Trait		Association effect^a^			*Hsp*RP ^b^		*Total (%)*
	Code	M	MxT	both	↑	↓	
Collar height	COL	8	11	1	13	7	*20 (11.3%)*
Peduncle Length	PdL	5	7	0	11	1	*12 (6.8%)*
Peduncle extrusion	PdE	4	5	1	10	0	*10 (5.6%)*
Seed Width	SdW	8	4	0	7	5	*12 (6.8%)*
Seed Length	SdL	2	5	2	6	3	*9 (5.1%)*
Seed Area	SdA	2	4	2	7	1	*8 (4.5%)*
Ear length	EAR	5	4	1	7	3	*10 (5.6%)*
Heading date	HEA	5	6	1	5	7	*12 (6.8%)*
Number of tillers	TILL	6	4	0	2	8	*10 (5.6%)*
Dry yield	DY	10	5	0	2	13	*15 (8.5%)*
Thousand Grain Weight	TGW	8	7	1	14	2	*16 (9.0%)*
Biomass yield	BY	5	3	1	4	5	*9 (5.1%)*
Harvest index	HI	8	8	2	1	17	*18 (10.2%)*
Grain per square meter	GSM	9	7	0	2	14	*16 (9.0%)*
	*Total (%)*	*85 (48.0%)*	*80 (45.2%)*	*12 (6.8%)*	*91 (51.4%)*	*86 (48.6%)*	*177 (100%)*

^a^Number of marker-trait associations identified for the marker main effect (M), the marker–treatment interaction (MxT) or both

^b^Effect of the wild barley alleles (*Hsp*) on the relative performance estimated for a trait: increased estimated mean (↑) or decreased estimated mean (↓) compared to the effect of the elite cultivar (*Hv*) performance

#### Plant developmental and morphological traits

The most numerous marker-trait associations were found for collar height (20 loci) with a generalised increased of the phenotypic means associated with the wild alleles. The QTLs COL10 (3H), COL14 (5H) and COL16 (6H) were identified as the most significant (*P* < 0.001) associations for the marker main effect (Additional file 6: Table S4). Each of these three loci explained a large proportion of the phenotypic variance increasing collar height by 27.1 cm (*R*^*2*^ = 30.5%), 14.5 cm (*R*^*2*^ = 31.2%) and 21.5 cm (*R*^*2*^ = 38.1%), respectively. Many of these associations were found to co-locate with major QTLs for closely related traits such as peduncle length and ear length. In contrast, the exotic alleles decreased plant height at 7 associated QTLs, the greatest observed at COL20 on chromosome 7H (10.73 cm shorter, *R*^*2*^ = 11.3%).

For heading date, 12 loci on 6 of the 7 chromosomes (none on 6H) were significantly associated with the trait (Additional file 6: Table S4). The exotic alleles contributed to reduce the estimated time to reach heading at seven of the marker-traits associations identified. The strongest associations (*P* < 0.001) for the trait were found on chromosomes 2H (HEA2, *R*^*2*^ = 51.1%) and 7H (HEA11, *R*^*2*^ = 46.8%) where the exotic alleles decreased the estimated time to reach heading by six days. At five marker-trait associations the exotic alleles were found to delay heading date, HEA4 on chromosome 2H delayed by 2.1 days (*R*^*2*^ = 10.8%). Similarly, the exotic genome was found to decrease the number of tillers at most of the QTLs defined for the trait. The largest effects were associated with TILL2 (2H, *P* < 0.001) and TILL8 (6H, *P* < 0.01) with 20.9 and 34.0 fewer tillers as compared to the elite barley alleles estimated effect. The latter was found concomitant with the chromosome region affecting plant height (COL16) and ear length (EAR9) on 6H.

#### Agronomic traits

Numerous marker-trait associations were found for agronomic traits including dry yield, biomass, harvest index and grains per m^2^ for which the wild barley alleles generally diminished the crop mean performance. For example, the exotic genome was found to reduce dry yield by up to 16.4% of the estimated yield for the elite alleles at DY3 on chromosome 2H (*R*^*2*^ = 23.7%) (Additional file 6: Table S4). A positive effect of the wild genome was only detected for minor QTLs such as DY4 (3H, *R*^*2*^ = 4.3%) and DY9 (4H, *R*^*2*^ = 6.8%), associated with yield increases of 16.3 and 11.6% under full irrigation respectively. Additionally, exotic alleles were found to slightly improve yield (2.7%) under drought conditions at DY8 (*R*^*2*^ = 3%) loci on 4H, however a 9.2% reduction under full irrigation was also associated with the locus. Similar results were obtained for chromosome regions associated with harvest index where maximum decrease on trait performance (from 42.6 to 35.5%) was associated with the mean effect of wild barley alleles at HI5 on 2H (*R*^*2*^ = 44.2%). This QTL collocated with DY3 on 2H (106.4 to 119.8 cM) (Additional file 6: Table S4).

In contrast, most QTLs defined for TGW were associated with positive effects of the exotic genome on the trait mean performance (14 out of 16 QTLs, 8 out of 9 positive associations at the marker main effect level). The wild alleles were found to increase grain weight up to 7.9% (i.e. 3.6 g) of the trait mean performance at TGW13 (5H) and up to 10.1% (i.e. 4.6 g) under partial irrigation at the minor association at TGW3 (2H) (Additional file 6: Table S4). Interestingly, some of the major associations found for the trait were concomitant with QTLs for seed parameters such as seed width. For example, an increase in 4.3% (i.e. 0.15 mm) of seed width associated to the wild genome was found for SdW11 in the same chromosome region of 5H for TGW13. Similar observations were made on chromosomes 3H for SdW5 and TGW8 (3.9 and 5.9% coincident increase on seed width and weight) and on chromosome 4H for SdW7 and TGW10 (2.9 and 4.9% increases respectively).

#### Pleiotropic effects of major developmental loci

**Fig. 3:**
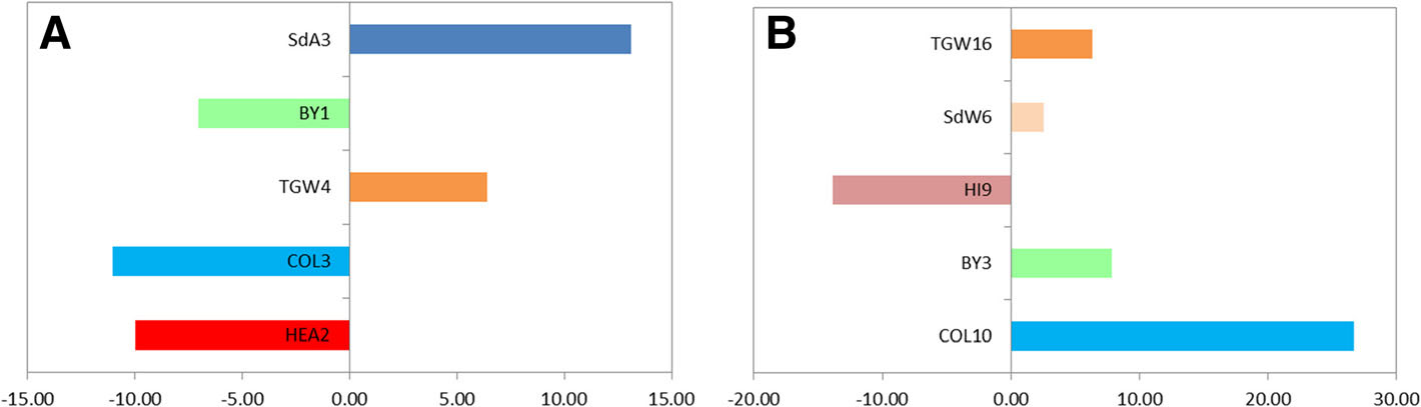
Pleiotropic effects of major developmental loci on agronomic performance in a group of QTLs clustering on chromosome 2H (**a**) and 7H (**b**). Bars correspond to the relative performance (%) of wild barley alleles on the trait (SdA: Seed area; DY: Dry yield, BY: biomass yield; TGW: Thousand grain weight; COL: collar height; HEA: heading date) in relation to the elite barley alleles. In both cases, heading date accounted for the strongest association at the loci (HEA2 and HEA11) explaining the two largest phenotypic variations found for the trait

Groups of QTLs were found to cluster at several genomic locations. Major associations for heading date and plant height were generally showing the strongest effect at these chromosome regions. For example, HEA2 on chromosome 2H (*R*^*2*^ = 51.1%, *P* < 0.001) was found to collocate with a major QTLs for biomass (BY1) as well as for collar height (COL3), peduncle length (PdL2) and seed parameters (SdL2 and SdA2) in the marker–treatment interaction effect (Fig. 3). Here the exotic alleles reduced time to heading by 6 days (*P* < 0.001), decreased biomass production (7% less, *P* < 0.001) and were shorter (up to 11.7 cm shorter plant under full irrigation, *P* < 0.01) but were associated with longer peduncles and larger seeds, particularly under drought (3.9 cm and 0.82 mm^2^ estimated increase respectively, *P* < 0.05). In addition, chromosome regions associated with TGW (TGW3 and TGW4) were found near HEA2 where the exotic alleles increased the mean trait performance by 6.4% (i.e. 2.9 g).

Similar effects were observed for a cluster of QTLs located on chromosome 7H sharing a peak marker region from 84.6 cM to 89.5 cM. Exotic alleles decreased time to heading by 6 days (HEA11) and reduced yield by 10.2% (DY14) while lengthening the plant peduncles PdE9 and PdL12 (4.4 cm and 4.9 cm respectively). The QTLs COL20, BY9 and TGW16 were found to collocate with the HEA11 locus (Fig. 3). Here the wild alleles were associated with 10.7 cm reduction on plant height, 911.5 kg ha^− 1^ less biomass yield and up to 3 g increase on TGW under drought respectively. Major developmental genes associated with plant phenology were thought to be responsible for the pleiotropic effect manifested across plant morphological and agronomic traits.

### Identification of candidates for grain weight and size improvement

In order to investigate putative candidate genes that could explain the phenotypic variation found for QTLs where wild alleles could contribute favourable alleles for improved crop performance, a few chromosome regions unlinked to developmental loci were targeted. The concomitant effect of seed weight and size was used as an exemplar to examine gene annotations of potential candidates that could be responsible for an increase in seed plumpness and therefore, seed size and weight.

The physical position of the flanking markers defining the SdW5/TGW8 locus on 3H and the SdW11/TGW13 locus on 5H defined a 3.6 Mbp and 2.9 Mbp regions, respectively, for which 92 and 51 annotated genes were found using the barley new genome assembly [27]. For the SdW5/TGW8 locus on 3H, the transcript HORVU3Hr1G010530.2 (corresponding to MLOC_61457.1 in the previous assembly [39] predicted to encode for a 14–3-3 protein was identified as an interesting candidate gene due to the regulative role of these proteins in the starch biosynthesis pathway in Arabidopsis [40] and during grain development in cereal crops such as wheat [41], rice [42] and barley [43]. In addition, HORVU3Hr1G010570.2 (MLOC_74351.2 in the previous assembly) was also targeted due to its function as an oligopeptide transporter (PTR2 family protein) and its potential role in the mobilisation of peptides in grain-filling seeds. Finally, for the SdW11/TGW13 locus on 5H, the HORVU5Hr1G093350.11 and HORVU5Hr1G093390.2 transcripts (MLOC_71738.1 and MLOC_76072.2 in the previous assembly respectively), both described as ‘Solute carrier family 22 member 1’, were identified as potential candidates for the locus.

## Discussion

Given the amount of valuable exotic genetic resource with potential for barley crop improvement and the need for enriching the crop genetic base for sustainable agriculture, effective pre-breeding strategies to systematically mine novel allelic variation are required [44]. In this study we genotyped, using the high throughput 9 K SNP chip [38], a representative set of 28 lines from a second backcrossed generation of RCSLs [9] to identify QTLs of agronomical relevance. Replicated and reproducible field trials in a semi-controlled and realistic environment, provided by a rain-out shelter, allowed precise phenotypic data for morphological, developmental and agronomic traits to be measured. In addition, we generated an improved genetic map for over 1800 SNP markers using the 9 K iSelect chip for barley [38], and designed an association analysis, by means of a mixed model approach, which allowed a coarse but accurate dissection of the genetics underlying quantitative and qualitative aspects of the crop. Putative candidate genes were identified using the recently published barley genome assembly [27].

### Improved wild genome marker coverage with a small number of representative lines

Using this approach we were able to achieve a greater degree of marker saturation, which improved the estimation of the extent and overlap of the substituted segments from the wild barley donor, doubling the number of markers mapped previously on the full set of 140 lines ([45]; 765 mapped SNPs). This meant that several traits, which had been observed but unmapped in a previous field evaluation [9] could be located to a specific position on the genetic map. For example, we were able to associate awn retention (an undesirable trait for cultivated barley) within a 5 cM region on chromosome 1H. Furthermore, the locus was found coincidental with the threshability locus *thresh-1* identified by Schmalenbach et al. [46] using a large set of 91 lines (BC_4_S_2_), providing similar mapping resolution as the 28 RCSLs used in our study. For some traits, however, the small sample size could only locate the QTL to large chromosomal regions. An example of this is the QTLs identified on 2H (HEA2) and 7H (HEA11), which were found to reduce heading date by 6 days when the wild barley allele is present. We identified the main determinant of long day photoperiod response locus (*Ppd-H1*) and an earliness per se locus (*eps7L*) [47] as good candidate genes for these major QTLs, respectively. However, the extent of the QTL effect spanned more than 40 cM in both cases, which could have partially masked the effect of other flowering genes in the proximity of these loci such as *HvCEN* on 2H [38] and the photoperiod response gene HvCO1 on 7H [48, 49]. Only by ‘breaking up’ the chromosome introgressions through new backcrosses in groups of Near Isogenic Lines (NILs) could some of these QTLs be located to smaller genomic regions.

### Detailed field phenotyping in a subset of 28 RCSLs evaluated under rain-out shelter

Most detailed phenotypic analyses required controlled experimentation and considerable replication which limits the number of lines that can be scored and is often unrepresentative of field conditions [50]. As a compromise we optimised the number of lines that represented the complete wild barley donor genome to perform detailed replicated and reproducible phenotyping in realistic semi-controlled trials under rain-out shelters over two consecutive seasons. The results obtained were supported by four replicates per genotype and treatment each growing season, despite the coarse estimation of QTLs derived from using fewer numbers of lines; we could achieve a good analytical power in the study. In addition, the experimental setup allowed an effective control over the water treatments while other environmental variables varied across the different treatments in the field trials. Moreover, monitoring the soil profile moisture throughout the two growing seasons and gathering climatic data from the onsite meteorological weather station was key in the understanding of the seasonal variations found for crop performance particularly for the irrigated treatments. An excess of irrigation delivered in the full irrigated plots could have caused hypoxia and waterlogging, having a negative impact on optimal crop production under irrigation. Nevertheless, establishing two irrigated regimes allowed us to define yield potential values in a non-stressed environment that could be used as a reference. This contrasts with previous QTL field studies of the entire RCSLs population, or a set of 80 lines, in which plants were grown in contrasting sites differing not only in annual rainfall (some under severe drought conditions) but also in soil characteristics, altitude and temperature [36, 51]. In previous studies, the scale of the experiments restricted the evaluations to a maximum of two replicates per genotype and season which could have limited the statistical power of the genotypic or environmentally - induced QTLs identified [52]. There are limitations to using small population sizes for QTL analyses and the power to detect small effect QTLs may be lost. The choice of population sizes has been discussed for decades and theoretically larger sizes are more likely to detect small effect QTLs, practically this is not always possible, and compromises are required. Yang et al. [53] addressed this question using a population of double haploids and conclude that with increased marker density, small populations were suitable for detecting QTLs. Because the introgressed regions are genetically well defined and almost the entire donor parent is represented, using a well characterised subset we can identify QTL to particular genomic locations. Validation and further fine mapping, using Near Isogenic Lines (NILs) is required to identify candidate genes for specific traits.

### RCSLs in the identification of putative candidate genes associated with the accumulation of spike cuticle waxes

Although the study was designed to identify quantitative traits, we were able to characterise and genetically dissect adaptive traits associated with the wild barley donor that had not been reported in previous studies of the RCSLs [9, 35–37, 51]. One such trait, which requires detailed observations, is spike glossiness. This is commonly associated with wild barley [16] and wild wheat [54]. The phenotype is caused by a depletion of β-diketones during the formation of spikes cuticle wax components which leads to a bright green non-glaucous phenotype due to modifications in the microcrystalline structure of the cuticular wax layer [55, 56]. Reduced glaucoussness increases tissues permeability to water loss, affects the angle at which irradiation is captured and increases tissues temperature, affecting the effective protection of the epicuticle as barrier against abiotic stresses such as drought or heat [42, 57]. From the literature, it is unclear the role that this phenotype would play in wild barley adaptation. We speculate that the increased wettability of non-glaucous spikes would favour seed imbibition and so seed germination in the water limited conditions typical from wild barley natural habitats. However, King and von Wettstein-Knowles [58] have shown that increased ear wetting could promote pre-harvest sprouting in humid environments and be detrimental to crop production. The segregation for spike glaucosity in the 28 RCSLs was localised to a 3.2 cM region between 0 and 3.2 cM on chromosome 1H for which wild alleles were associated with the glossy non-glaucous phenotype. The locus seems to correspond to the eceriferum gene locus *Cer-yy* reported by Lundqvist and Von Wettstein-Knowles [59] for which no candidate genes have been found. In our study, we identified three putative genes for the locus based on their annotations and their conceivable function in the wax biosynthesis pathway taking other *cer* mutants described for barley as a reference [54, 60]. An esterase/lipase/thioesterase family protein, an O-acyltransferase (WSD1-like) family protein and a fatty acyl-CoA reductase were tagged as potential candidates involved in the generation and transport of fatty acids conforming the wax cuticle [55, 60, 61]. However, it is interesting to note that, because of the dominant inheritance of the gene, the function of *Cer-yy* has been described as an active regulator in the differentiation process of spike lemmas rather than in the process of wax synthesis and deposition by itself [62]. Therefore, other candidate genes that might be involved in the regulation of events occurring during spike differentiation would need to be considered. Investigations are underway to fine map the locus and elucidate its function in sets of NILs differing for the *Cer-yy* locus.

### Major developmental loci determined the largest phenotypic variations found in the quantitative variation of traits

The strongest marker-trait associations found were related to known major developmental genes such as *Ppd-H1* and *eps7L* flowering genes and the *sdw1-denso* semi-dwarfing mutation. Yield related QTLs cluster around these loci due to the strong pleiotropic effects that flowering has on plant performance. In most of the cases, the introgression of exotic alleles at these loci limit vegetative growth or increase plant height, which in general negatively affect traits such as grain yield and harvest index. The effect agrees with observations in other backcross populations using germplasm or landraces from the Fertile Crescent as donor genome [22, 33, 63–66] which, to some extent, reflects the strong selective pressures that these traits have experienced during domestication and particularly through the crop breeding process [6, 67–69]. Interestingly, however, TGW, which is often used as a measure of yield, was shown to be greater in most of the RCSLs, an observation also noted by Matus et al. [9]. Several of the marker-trait associations for thousand grain weight (TGW4 on 2H and TGW16 on 7H) appear coincidental with QTLs identified using the larger population of 137 RCSLs genotyped with 1536 SNP markers [70] and phenotyped in contrasting Chilean environments over one growing season [51]. In our study, the wild alleles increased grain weight by 6.4% for the marker main effect at TGW4 and in response to water stress at TGW16. However, it is most likely that these are determined by the pleiotropic effects of the major developmental loci, HEA2 and HEA11, in the region for *Ppd-H1* and *eps7L* flowering time genes.

### Potential candidate genes associated with transport and accumulation of carbohydrates were identified for locus where Caesarea 26–24 alleles contributed gains in grain size and weight of the elite cv. Harrington

Manipulating source-sink relationships and deciphering the genetics and physiological mechanisms associated with dry matter partitioning in grain development is one of the major targets for achieving significant genetic gains in yield of major cereal crops [71, 72]. Genes associated with effective transport of sucrose [73] into the growing grain as well as those responsible for cell growth and expansion [74] and carbohydrates metabolism during grain development [75] have been directly associated with modifications on grain weight and its components (grain length and width) and therefore, potentially useful for optimising grain development in cereal crops. However, the segregation for main developmental loci seems to largely interfere in the localisation of loci associated with stable variations on grain weight and size in QTL studies in biparental populations [76, 77]. In our study we were able to identify two relatively small non-developmental loci on chromosomes 3H and 5H where the wild alleles were associated with a 5.9 and 7.9% increase in main grain weight, respectively (i.e. mean additive effects of 2.7 g and 2.2 g over 1000 grains respectively), showing concomitant positive effects on grain width. Evidence from the literature pinpointed the region on 3H as a particularly interesting target to investigate in the future. We identified an interesting potential candidate gene which encodes a 14–3-3 protein reported to be actively involved in the regulation of the carbohydrate metabolism in barley, predominantly during grain filling and germination [43, 78]. Indeed, Alexander and Morris [43] showed how increased levels of 14–3-3 protein in barley endosperms inhibited the activity of sucrose synthase proteins responsible for the storage of starch in sink tissues. Similar roles for these proteins have been reported in wheat [41] and rice [79] grain development for which accumulation of 14–3-3 transcript and formation and accumulation of starch were negatively correlated leading to poorly filled grains. In addition, 14–3-3 proteins have also been found as regulators in the formation of trehalose 6 phosphate (T6P), an intermediate compound in the trehalose biosynthesis pathway [80, 81]. This key molecule acts as a signal for sucrose availability having a huge influence on the stimulation and rate of starch biosynthesis in plant sink tissues [82–84] which is particularly important in the development of the starchy endosperm during grain development of cereal crops such as wheat [75]. Therefore, despite the synthesis of polysaccharides in cereals endosperm being rather intricate, we could hypothesise the exotic allelic variant for this QTL contributes to differences in the elite barley sink strength or the ability to accumulate photo-assimilates in the grain which could contribute constitutive genetic gains in crop yield performance. This hypothesis was hinted at in previous studies, suggesting genotypic differences in the translocation and accumulation of carbohydrates and osmolytes in the RCSLs [37, 85], however further investigations using NILs for the targeted regions of these QTL to fine map or transcriptomic analysis on groups of RCSLs harbouring the introgression on 3H would be necessary to support this hypothesis.

The utilisation of the new barley genome assembly [27] was key in the identification of interesting candidate genes explaining the phenotypic variation associated with these loci. However, it should be noted that the abundance of marker–trait associations detected (177 in total) could represent some overestimations that may confound the results of the analysis possibly as a consequence of epistatic interaction between wild barley introgressions [86, 87]. Nevertheless, we see our approach as a useful first step towards the genetic dissection of traits, which require detailed phenotyping and replication as well as those that vary across seasons. Indeed, favouring greater replications and precision phenotyping in small but genetically well-characterised sets of lines has been seen as an effective optimisation for QTL-mapping rather than increasing the number of lines evaluated at the expense of replicates [53]. More and more novel secondary traits with direct impact on crop production and sustainability appear to be interesting targets for breeding programmes, however little is known about their genetic control given that, in many cases, their evaluation requires sophisticated or costly evaluations less suitable for large mapping population. For instance, rhizodeposition and soil microbial interactions are complex traits which have been found to affect soil C content and availability of water and nutrients for plants. Recently, Mwafulirwa et al. [88], showed variability in soil carbon dynamics associated with genotypic differences in a small group of six RCSLs used in the present study. In this first evaluation no marker-trait association study could be conducted, however the principle of the current study could be useful for identification of marker-trait associations in a manageable group of lines. Similarly, genotypic differences for traits associated with improved photosynthetic performance under severe drought conditions such as grain Δ^13^C, have been reported in small groups of RCSLs [37]. These differences could eventually relate to genotypic differences in root system development across the population. In fact, in a recent evaluation of few RCSLs we found important genotypic variations of root growth parameters in a 2D pouch experimental approach [89]. Hence, precise phenotyping of these traits in the subset of 28 RCSLs could lead to the identification of major loci governing phenotypic variation of new interesting traits requiring thorough and expensive analysis. However, it is important to keep in mind that only genetic dissection of highly heritable quantitative traits might be achievable [53] and that for increasing the accuracy of the QTL detection and reduce the number of misleading associations more RCSLs would need to be included in the study.

## Conclusions

Here we have proposed an approach to dissect quantitative traits using an exotic genetic library selected from an early backcrossed generation (BC_2_). A single locus analysis by means of a mixed model approach was found effective for coarse QTL location in a reduced group of recombinant lines with more than one alien introgression per line and/or chromosome. We were able to identify stable marker trait associations for relevant agronomic characters that were compared with the literature and previous QTL studies in the RCSLs [51]. Most of the QTLs clustered at developmental loci where exotic alleles had a strong effect on plant phenology and development. Nevertheless, non-developmental loci associated with favourable effects of the exotic genome on crop performance could also be identified.

Finally, wild barley accessions are being recognised as a novel source of allelic variation, which could contribute to enhance crop performance under challenging environments [3, 4]. In addition to introgession lines using wild barley as a donor, other advanced backcross populations are being developed together with state-of-the-art genomics technologies will provide that step change required using this yet untapped variation.

## Methods

### Plant material and genotypic characterisation

Twenty-eight Recombinant Chromosome Substitution Lines (RCSLs) were selected to represent the entire genome of the Israeli wild barley accession, Caesarea 26–24, in the uniform elite genetic background of the North American two-row spring malting barley cv. Harrington [9]. Briefly, the lines were obtained through an advanced backcross strategy [90] by which, after two backcrosses with the recurrent parent (cv. Harrington) and six generations of self-pollination (BC_2_F_6_), the wild donor genome was segmented and introgressed in 137 RCSLs [9]. The selection of a subset of 28 RCSLs was based on the genotypic architecture of the lines and a minimum tilling panel for each of the barley chromosomes using SNP marker data generated as part of a Generation Challenge Programme [45] for the Barley Oligo Pool Assay 1 (BOPA1) [70]. For the present study, the set of preselected RCSLs was advanced to the BC_2_F_9_ generation and characterised for a larger set of markers from 9 K Infinium iSelect SNP platform (7864 gene-based SNP markers) described by Comadran et al. [38].

Genomic DNA was extracted from leaf samples of ten-day-old seedlings (around 100 mg wet weight plant material) from the 28 RCSLs, cv. Harrington and Caesarea 26–24 using the QiagenDNeasy Plant Mini Kit (Qiagen). Genotyping was conducted by TraitGenetics GmbH (Gatersleben, Germany) using DNA samples at a concentration of 50 ng μl^− 1^. The genotype calls were analysed using the Illumina GenomeStudio Genotyping (GT) module (Illumina, San Diego, CA) and Flapjack [91]. Polymorphic SNP marker data was imported to Graphical Genotypes software, GGT 2.0 [92], to visualise and characterise the proportion of exotic chromosomal regions from the donor parent introgressed into the elite genetic background for each of the lines.

### Field experimental set-up and phenotypic characterisation

**Fig. 4:**
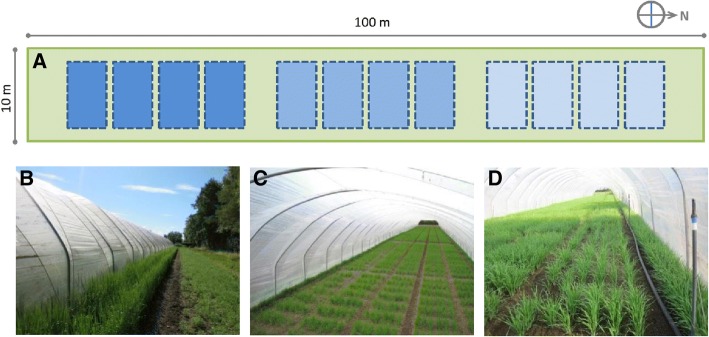
Field experimental setup. **a** Schematic layout of the row column experimental design with three water treatments superimposed on top and four replicates (doted boxes) within each water treatment. Droughted (light blue) and fully irrigated (dark blue) plots faced north and south respectively in 2013. This arrangement was swapped in 2014. Partially irrigated plots (medium blue) were established in between the other two water treatments. Guard plots (green) were sown between water treatments and the edges of the rainout shelter. **b** Guard plots view from along the outer long edge of the rain-out shelter. **c** Top view of the field trial. **d** Sprinkler irrigation system

The 28 RCSLs and the recurrent parent, cv. Harrington, were grown in field trials over two growing seasons (2013 and 2014) at The James Hutton Institute (Latitude 56.45°N, Longitude 3.06°W). In order to characterise the RCSLs under different water regimes, the experiments were carried out using a rain-out shelter that protected the plots subjected to water deficit from rainfall providing better control over the soil water content in the irrigated treatments. The experiments were established in a row column design with water treatments and replicates superimposed. Four replications and three water treatments were established each growing season at different location within the field trial. Experimental plots were arranged in 72 rows and 5 columns along the trial. Each plot consisted of six 0.8 m long rows of plants with a 0.2 m gap between plots. Guard plots (cv. Concerto) were sown in extra rows between the replicates within each water treatment and along the sides of the trial to minimize the edge effect. In addition, an extra row of guard plots was sown at the opened ends of the rain-out shelter and along the sides of the field trial to reduce the amount of water coming from rainfall (Fig. 4).

Sowing dates were 15th and 17th April in 2013 and 2014, respectively. Seeds were sown using a Wintersteiger Seedmatic drill at 4 cm depth. In 2013 the granulated fertilizer HYDRO Sulphur cut (22% N, 4% P_2_O_5_, 14% K_2_O, and 7.5% SO_3_) was used as a seed dressing and in 2014 as a top-dress fertilizer. The fertilizer was applied at 270 kg ha^− 1^ the day of sowing and up to 500 kg ha^− 1^ a week after sowing following the local agronomic practices for spring barley. To minimize the effect of pests, field trials were treated with fungicides Bravo 500 (1 l ha), SiltraXPro (0.6 l ha^− 1^), Vegos (0.25 l ha^− 1^) and Justice (0.15 l ha^− 1^) on the 7th and 9th June and for aphids on 11th and 17th July in 2013 and 2014, respectively. Once the seedlings had established in the field at growth stages GS13-GS14 [93], the rain-out shelter was built and skinned with polythene film 0.15 mm thick Clear-High UV (Visqueen, UK). This occurred about a month after sowing, the 16th May 2013 and 14th May 2014, when the sprinkler irrigation system was set up defining different water treatments from the 23rd May and 21st May in 2013 and 2014, respectively.

#### Water treatment

Two irrigated (full irrigation and partial irrigation) and non-irrigated treatments (drought) were established to assess the response to water deficit in the development of the crop. The irrigated plots were watered at the equivalent of 2.5 mm rainfall per day based on the Met Office rainfall average records for east Scotland from 1910 during the barley growing season (http://www.metoffice.gov.uk/). The irrigation was stopped at different growth stages [93] in these two treatments: at grain milk development (GS 73–77) in fully irrigated plots (23rd July in 2013 and the 24th July in 2014) and before anthesis (GS 50–55) in the partially irrigated plots (24th June in 2013 and 23rd June in 2014) and no more water was added to the plots. The non-irrigated plots constituted the drought treatment and they were not watered after the rain-out shelter was built.

The soil moisture content in the field was monitored on a weekly basis using a capacitance probe PR2/4 (Delta-T Services Ltd) at different depths within the soil profile in 53 access tubes evenly distributed across the field trial. In addition, meteorological data were obtained from The James Hutton Institute weather station records. Daily air and soil temperature and rainfall accumulation were collated to define the climate conditions prior to the setup of the rain-out shelter and throughout the field experiments in each growing season.

#### Phenotypic traits measured

The RCSLs were characterised for thirteen morphological, developmental and agronomic traits in the 2013 and 2014 field trials (Table 1). Additionally, qualitative traits such as seed shattering, grain threshability, purple grain and glossy spike were recorded as presence or absence throughout crop development. At plant physiological maturity, the two middle rows of each experimental plot were hand-harvested and used to determine yield and yield component data for each genotype.

### Data analysis

#### Phenotypic variation of quantitative traits

Residual maximum likelihood method (REML) was used to estimate fixed effects and random effect parameters in the traits measured [94]. A three factorial mixed model analysis was used to assess the effect of genotype, treatment, growing season and their interaction on the phenotypic variation observed in thirteen quantitative traits. Statistical significance for the fixed model effects was assessed by using a chi-squared based Wald-test. The random term included the replicate and the column to take into account the spatial variation in the field trials. Therefore, in a first approach the marker information was not included, and the genotypes were considered in the fixed term of the three factorial mixed model analysis that is presented in the following section.

In order to identify genotypes that significantly differed from the recurrent parent, a Fisher’s least significance difference (LSD) test was used to compare the Best Linear Unbiased Estimators (BLUEs) of the fixed terms at 0.01 levels of probability. The VMCOMPARISON procedure in Genstat 17 (VSN International, UK) was computed for the analysis.

#### Marker-trait association analysis

Marker-trait associations for qualitative measurable traits were visually identified as overlapping exotic chromosome introgressions shared among RCSLs showing the wild phenotype. For quantitative traits, identification of associations required the development of an analytic approach that integrated the molecular marker information into a mixed model to test the effect of SNPs on the phenotypic variation. The estimates for fixed and random parameters of the mixed model were obtained by residual maximum likelihood (REML) method [94] using GenStat 17th Edition (VSN International, UK). The trait response was calculated according to the following hierarchical model:

1 $$ {Y}_{ijklmno}=\mu +{M}_i+{T}_j+{M}_i\ast {T}_j+{Y}_k+{M}_i\ast {Y}_k+{T}_j\ast {Y}_k+{R}_l\left({T}_j\ast {Y}_k\right)+{C}_m\left({T}_j\ast {Y}_k\ast {R}_l\right)+{G}_n+{\varepsilon}_{o(ijklmn)} $$

where *μ* is the general mean, M_*i*_ is the fixed effect of the *i*-th SNP marker, T_*j*_ is the fixed effect of the *j*-th water treatment, M_i_*T_*j*_ is the fixed effect of the interaction of the *i*-th SNP marker and the *j*-th treatment, Y_*k*_ is the fixed effect of the *k*-th year, M_i_*Y_*k*_ is the fixed effect of the interaction of the *i*-th genotype and the *k*-th year, T_j_*Y_*k*_ is the fixed effect of the interaction of the *j*-th treatment and the *k*-th year, R_*l*_(T_j_*Y_*k*_) is the random effect of the *l*-th replicate nested in *j*-th treatment and *k*-th year, C_*m*_(T_j_*Y_k_*R_*l*_) is the random effect of the *m*-th column nested in the *j*-th treatment, *k*-th year and *l*-th replicate, G_n_ is the random effect of the *n*-th genotype and ε_*o(ijklmn)*_ is the residual term of X_*ijklmno*_*.* The analysis was conducted for one marker at a time in a loop computed for each trait.

For the analysis, blocks of contiguous markers that were polymorphic for the same RCSLs were treated as a single entity. Since the wild barley introgressions overlapped across contiguous regions within a chromosome, the length of each genomic region tested was determined as the genetic distance between the first SNP markers defining adjacent loci. This simplified the computational process of the analysis by removing redundant marker information. Nevertheless, mapping information of the entire set of markers was taken into account to define the size of the chromosome regions associated with the phenotype observed or QTLs.

#### QTL location and identification of candidate genes

Blocks of markers with significant main effects and/or interactions with the water treatment at 0.05(*), 0.01(**) and 0.001(***) levels of significance were considered for QTL location. Neighbouring blocks of markers showing significant effects in the same direction were assumed to be part of the same QTL and used to define the significant region of the marker–trait association. The *p* value of the most strongly associated SNP marker–trait within a chromosome region was used to define the peak region of the QTL. Since we were interested in identifying only stable marker-trait association across experiments and treatments, the SNP × Year interaction were not investigated further.

The Best Linear Unbiased Estimates (BLUEs) for each trait for the donor ([*Hsp*]) and the recurrent parent ([*Hv*]) alleles obtained for the significant peak region were used to calculate the relative contribution (RP) of the exotic parent alleles on the trait performance as follows:

2 $$ RP\left(\%\right)=\frac{\left[ Hsp\right]-\left[ Hv\right]}{\left[ Hv\right]}\ast 100 $$

The position of major determinant genes related to plant phenology and morphology was estimated from previous studies and public gene databases to support the results obtained from this analysis. Additionally, some genomic regions accounting for a significantly large proportion of a trait’s variance (R^2^) were used to identify possible putative candidate genes underlying the predicted phenotypic variation. In this case the number of genes in the targeted regions was first estimated by looking at the physical position of the iSelect SNP markers in the newly released barley genome assembly [27]. Then putative gene content was compiled by examining the annotated high-confidence genes for each defined region. Finally, possible candidates were identified by gene ontology annotations and the homologies found in other crop relative species genomes using Basic Local Alignment Search Tool (BLAST) analysis on the NCBI website.

#### Stability analysis of agronomic traits

Values for yield (DY) and grain weight (TGW) for each of the RCSLs were used to investigate the effect of the exotic genome in the performance and stability of the crop across the environmental conditions imposed in the field. Genotype by environment (GE) interaction was studied by means of an additive main effects and multiplicative interaction model (AMMI) for which six environments were defined as the combination of growing season and water treatment. Using this method, the overall variation observed for yield traits such as dry yield and thousand grain weight was partitioned into genotype main effects, environment main effects and GE interaction [95]. The model combines a principal component analysis (PCA) of the GE interaction that is obtained as a result of a two factorial analysis of variance taking genotype and environment as the main effect. The AMMI model is:

3 $$ {Y}_{GE}=\mu +{G}_i+{E}_j+\sum \limits_{k=1}^n{\lambda}_k{\gamma}_{ik}{\delta}_{jk}+{\rho}_{ij}+{\varepsilon}_{ij k} $$

where Y_GE_ is the yield value of genotype G on environment E, *μ* is the general mean, G_i_ is the genotype effect, E_j_ is the environment effect, λ_k_ is the singular value (eigenvalue) of the k-th principal component axis, γ_ik_ and δ_jk_ are the genotype and the environment scores (eigenvectors) for the k-th principal component axis, ρ_ij_ is the interaction residual and ε_ijk_ is the random error.

As a result, the interaction principal components generated (IPCA1 and IPCA2) are used to graphically summarise (biplots) the GE variation observed. The AMMI2 biplot uses the genotypes and environments scores for the first two IPCA components (IPCA1 on the X-axes and IPCA2 on the Y-axes) giving information about the GE patterns observed. Genotypes with IPCA scores close to zero are more stable or widely adapted to the tested environments whereas specific adaptation of the genotypes is determined by the length of the orthogonal projection of the genotype points onto the environmental vectors. In addition, the AMMI1 biplot tests the genotypes yield potential and stability simultaneously by plotting in the same diagram the average yields (X-axes) and the first dimension measure of GE interaction (IPCA1) for both genotypes and environments (Y-axes). AMMI model was computed using Genstat 17 (VSN International, UK).

## Additional files

Additional file 1: **Table S1.** RCSLs genotypes determined for 1848 SNP markers from the 9 K SNP chip for barley [38]. (XLSX 408 kb)

Additional file 2: **Figure S1.** Climate data before and throughout the field trial in (A) 2013 and (B) 2014. Accumulated rainfall (mm) values per week (x axes), air maximum and minimum average temperature values per week (± SE). Data obtained from James Hutton Institute weather station (56.45°N; 3.07°W). Field trials were established in the week 16 both years. (DOCX 28 kb)

Additional file 3: **Figure S2.** Volumetric water content (ml cm^− 3^) in the soil profile at (A) 100 mm, (B) 200 mm, (C) 300 mm and (D) 400 mm depth in the full irrigated (blue), partial irrigated (green), and drought (red) water treatment in 2013 (left) and 2014 (right) field trials. Vertical dashed lines indicate beginning and end of heading time referred to days after sowing (DAS) for year. Error bars indicate standard error of the mean. (DOCX 258 kb)

Additional file 4: **Table S2.** Climate data before sowing, during seedling establishment, before heading and during heading in the 2013 and 2014 growing seasons. Air and soil mean temperature values (± SE) and accumulated rainfall (mm) values obtained from the James Hutton Institute weather station (56.45°N; 3.07°W). (DOCX 15 kb)

Additional file 5: **Table S3.** Genotypic mean values (± SE) for the morphological, developmental and agronomic traits registered in 2013 and 2014 field trials. Genotypes are arranged in ascending order from top to bottom of the table for collar height (COL), peduncle length (PdL), ear length (EAR), seed area (SdA), seed length (SdL), seed width (SdW), heading date (HEA), number of tillers (TILL), dry yield (DY), thousand grain weight (TGW), biomass yield (BY) and harvest index (HI). Best linear unbiased estimators (BLUEs) for genotypes labelled with different letters differ significantly (*P* < 0.01, Fisher’s LSD multi- comparison test). Harrington mean values highlighted in bold. (XLSX 20 kb)

Additional file 6: **Table S4.** Significant quantitative trait locus (QTLs) detected on the RCSLs exotic genetic library associated with thirteen morphological, developmental and agronomic traits. One trait per tab. (XLSX 167 kb)

## Abbreviations

AB: Advanced backcross
AMMI: Multiplicative Interaction Model
BLUEs: Best Linear Unbiased Estimators
BY: Biomass yield
COL: Collar Height
DY: Dry yield
EAR: Ear Length
GLS: Spike glossiness
HEA: Heading date
HI: Harvest Index
LOD: Lodging
PCA: Principal Component Analysis
PdE: Peduncle Extrusion
PdL: Peduncle Length
QTL: Quantitative Trait Loci
RCSLs: Recombinant Chromosome Substitution Lines
REML: Residual maximum likelihood method
RILs: Recombinant Inbred Lines
SdA: Seed Area
SdL: Seed Length
SdW: Seed Width
SHT: Seed shattering
SNPs: Single nucleotide Polymorphisms
TGW: Thousand grain weight
THR: Grain threshability
TILL: Number of Tillers
