# Supplementary material for: Genetic dissection of quantitative and qualitative traits using a minimum set of barley Recombinant Chromosome Substitution Lines
Source: BMC Plant Biol. 2018 Dec 7;18:340. doi: 10.1186/s12870-018-1527-7 (PMC6286510; doi:10.1186/s12870-018-1527-7)
Supplement: Supplementary file 2 — Figure S1. Climate data before and throughout the field trial in (A) 2013 and (B) 2014. Accumulated rainfall (mm) values per week (x axes), air maximum and minimum average temperature values per week (± SE). Data obtained from James Hutton Institute weather station (56.45°N; 3.07°W). Field trials were established in the week 16 both years. (DOCX 28 kb) [file 12870_2018_1527_MOESM2_ESM.docx]

**Additional file Figure S2 (continued). Climate data before and throughout the field trial in (A) 2013 and (B) 2014** Accumulated rainfall (mm) values per week (x axes), air maximum and minimum average temperature values per week (± SE). Data obtained from James Hutton Institute weather station (56.45°N; 3.07°W). Field trial were established on the week 16 both years.
