# Supplementary figures and images for: Genetic dissection of quantitative and qualitative traits using a minimum set of barley Recombinant Chromosome Substitution Lines
Source: BMC Plant Biol. 2018 Dec 7;18:340. doi: 10.1186/s12870-018-1527-7 (PMC6286510; doi:10.1186/s12870-018-1527-7)

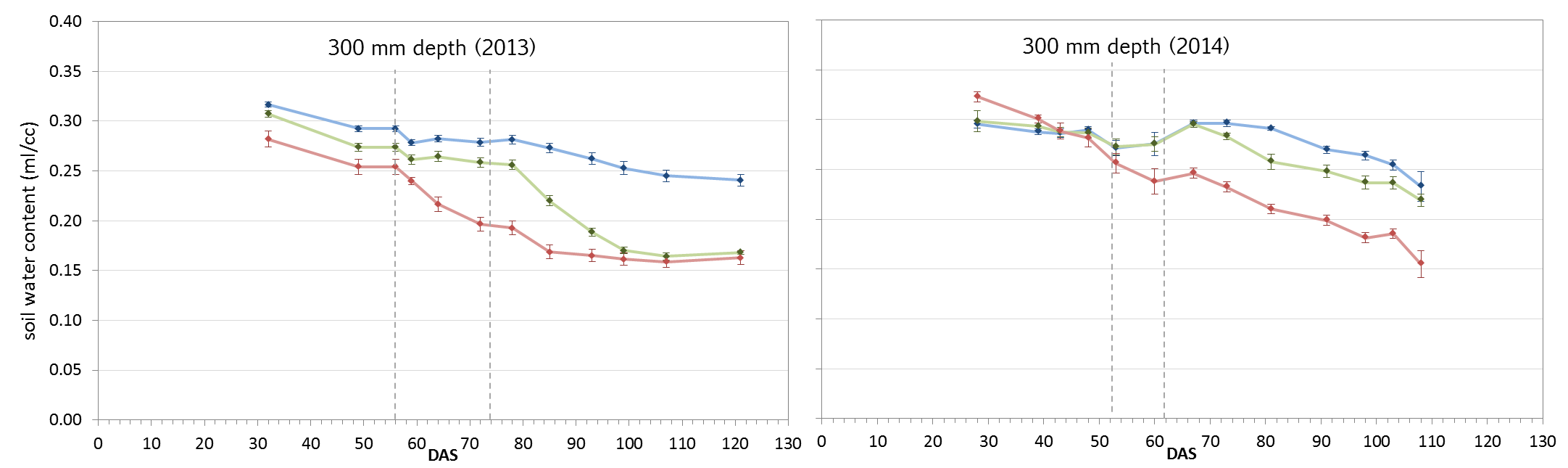

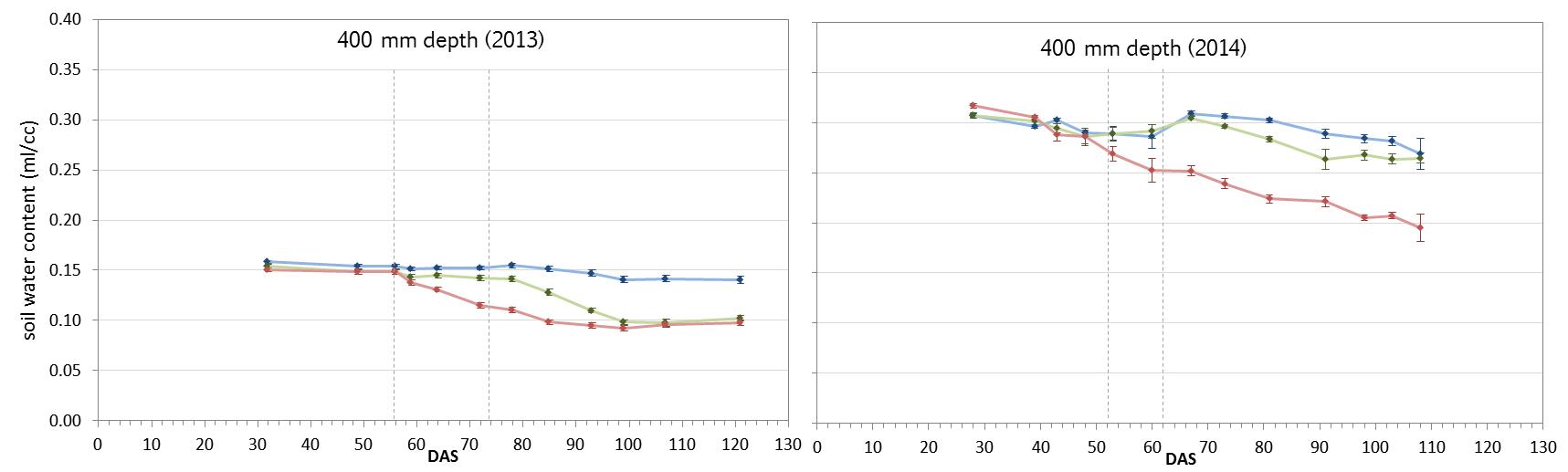

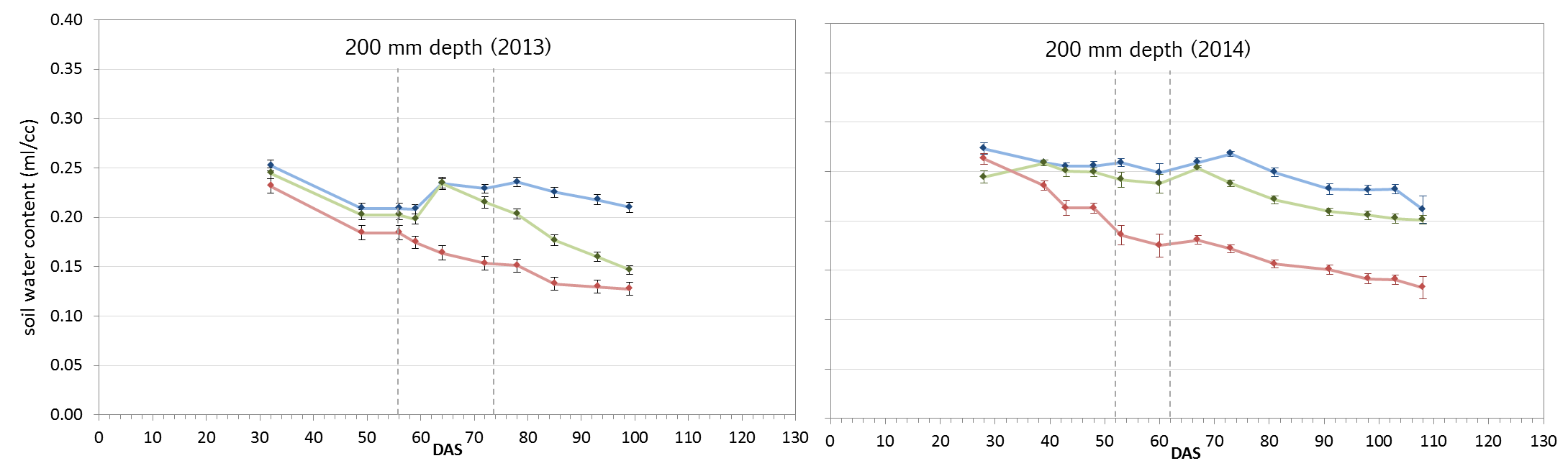

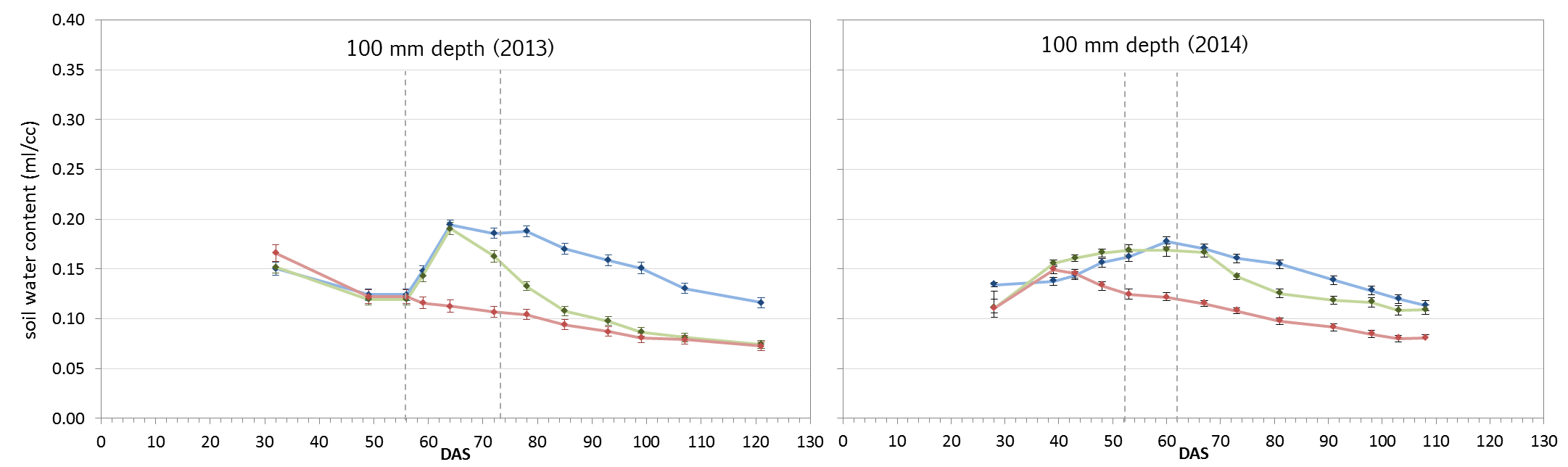


D

C

B

A

Supplement: Supplementary file 3 — Figure S2. Volumetric water content (ml cm− 3) in the soil profile at (A) 100 mm, (B) 200 mm, (C) 300 mm and (D) 400 mm depth in the full irrigated (blue), partial irrigated (green), and drought (red) water treatment in 2013 (left) and 2014 (right) field trials. Vertical dashed lines indicate beginning and end of heading time referred to days after sowing (DAS) for year. Error bars indicate standard error of the mean. (DOCX 258 kb) [file 12870_2018_1527_MOESM3_ESM.docx]
