# Supplementary material for: Genetic dissection of quantitative and qualitative traits using a minimum set of barley Recombinant Chromosome Substitution Lines
Source: BMC Plant Biol. 2018 Dec 7;18:340. doi: 10.1186/s12870-018-1527-7 (PMC6286510; doi:10.1186/s12870-018-1527-7)
Supplement: Supplementary file 4 — Table S2. Climate data before sowing, during seedling establishment, before heading and during heading in the 2013 and 2014 growing seasons. Air and soil mean temperature values (± SE) and accumulated rainfall (mm) values obtained from the James Hutton Institute weather station (56.45°N; 3.07°W). (DOCX 15 kb) [file 12870_2018_1527_MOESM4_ESM.docx]

Supplementary Material 2. Table S2 Climate data before sowing, during seedling establishment, before heading and during heading in the 2013 and 2014 growing seasons. Air and soil mean temperature values (± SE) and accumulated rainfall (mm) values obtained from the James Hutton Institute weather station (56.45°N; 3.07°W).

|  |  | Mean temperatures in ^o^C (± SE) | | |  |
| --- | --- | --- | --- | --- | --- |
| **Time period**  *Year* | Date  (No days) | Air Max  (min–max) | Air min  (min–max) | Soil ± SE  (min–max) | Rainfall^1^  (days) |
| **Before sowing** |  |  |  |  |  |
| *2013* | 01 Jan – 15 Apr | 6.03 ± 0.26 | 0.43 ± 0.22 | 2.15 ± 0.16 | 216.5 |
|  | (105 days) | (1.60 – 13.90) | (-5.00 – 8.80) | (-1.20 – 7.20) | (66) |
| *2014* | 01 Jan – 17 Apr | 9.17 ± 0.26 | 2.80 ± 0.25 | 4.38 ± 0.19 | 230.6 |
|  | (107 days) | (4.70 – 15.70) | (-2.50 – 7.80) | (0.70 – 9.00) | (78) |
| **Seedling establishment** |  |  |  |  |  |
| *2013* | 16 Apr – 16 May | 12.63 ± 0.46 | 4.63 ± 0.42 | 7.92 ± 0.32 | 63.7 |
|  | (31 days) | (3.80 – 17.70) | (1.60 – 8.70) | (2.80 – 11.40) | (18) |
| *2014* | 18 Apr – 14 May | 13.83 ± 0.43 | 5.79 ± 0.58 | 10.44 ± 0.30 | 60.3 |
|  | (27 days) | (9.80 – 17.20) | (-0.70 – 9.90) | (7.20 – 12.80) | (22) |
| **Before heading** |  |  |  |  |  |
| *2013* | 15 Apr – 07 Jun | 14.42 ± 0.46 | 5.51 ± 0.38 | 9.82 ± 0.41 | n/a |
|  | (54 days) | (3.80 – 21.40) | (1.60 – 12.40) | (2.80 – 17.60) | n/a |
| *2014* | 17 Apr – 06 Jun | 15.31 ± 0.39 | 7.24 ± 0.44 | 12.01 ± 0.31 | n/a |
|  | (51 days) | (9.80 – 20.50) | (-0.70 – 12.80) | (7.20 – 16.10) | n/a |
| **During heading** |  |  |  |  |  |
| *2013* | 08 Jun – 25 Jun | 17.27 ± 0.39 | 8.93 ± 0.48 | 15.02 ± 0.33 | n/a |
|  | (18 days) | (14.20 – 20.10) | (5.90 – 12.10) | (12.50 – 17.00) | n/a |
| *2014* | 07 Jun – 17 Jun | 19.56 ± 0.45 | 10.97 ± 0.47 | 15.93 ± 0.27 | n/a |
|  | (11 days) | (18.30 – 23.80) | (8.30 – 13.70) | (15.00 – 18.30) | n/a |
